# High-Fructose-Induced Salt-Sensitive Hypertension: The Potential Benefit of SGLT4 or SGLT5 Modulation

**DOI:** 10.3390/nu17152511

**Published:** 2025-07-30

**Authors:** Sharif Hasan Siddiqui, Noreen F. Rossi

**Affiliations:** 1 Department of Physiology, Wayne State University, Detroit, MI 48201, USA; hq4700@wayne.edu; 2 John D. Dingell VA Medical Center, Detroit, MI 48201, USA

**Keywords:** fructose, salt, diet, SGLT4, SGLT5, prevention, hypertension

## Abstract

Hypertension is an important risk factor for cardiovascular diseases. High salt intake when consumed with excess fructose enhances hypertension and resultant cardiovascular disease. Usually, the small intestine absorbs dietary fructose, and the proximal tubule of kidney reabsorbs filtered fructose into the circulation with the help of different transporters including SGLT4 and SGLT5. Very recently, SGLT5 mRNA has also been found to be expressed in the heart. High-fructose diet stimulates the sympathetic nervous system and renin–angiotensin–aldosterone (RAAS) activity, of which both are responsible for endothelial dysfunction and are associated with salt-sensitive hypertension. Few studies exist regarding the effects of SGLT4 and SGLT5 on cardiovascular function and blood pressure. However, SGLT4 gene knockout does not alter fructose-associated impact on blood pressure. In contrast, blood pressure does not increase in SGLT5 knockout rats even during fructose consumption. Given that limiting fructose and salt consumption as a public health strategy has proven challenging, we hope that studies into SGLT4 and SGLT5 transporters will open new research initiatives to address salt-sensitive hypertension and cardiovascular disease. This review highlights current information about SGLT4 and SGLT5 on fructose absorption, salt-sensitive hypertension, cardiovascular disease and points the way for the development of therapeutic fructose inhibitors that limit adverse effects.

## 1. Introduction

Fructose is a six-carbon monosaccharide naturally found in fruits, honey and some vegetables; therefore, it is sometimes known as “fruit sugar.” Fructose along with glucose is a component of the widely used disaccharide, sucrose [1]. Since its introduction as high-fructose corn syrup (HFCS) in 1967, fructose has been widely incorporated in processed foods and beverages. Fructose is ~1.7 times sweeter than sucrose and about twice as sweet as glucose. Moreover, the cost of HFCS is a fraction of that for either sucrose or glucose. The combination of high sweetness and low cost makes fructose highly profitable as an additive [2].

Despite its low glycemic index, fructose has been implicated as a substantial contributor to a number of different metabolic diseases including obesity, hepatic steatosis, insulin resistance, frank diabetes mellitus, hypertriglyceridemia, uric acid over-production, chronic kidney disease, and cardiovascular dysfunction [3,4,5,6,7]. Excess fructose consumption over longer periods of time has been associated with cardiac fibrosis, diminished arterial compliance increased glomerular mesangial matrix formation, and decreased glomerular filtration rate [8]. These fructose effects on the heart, vasculature, and kidney play important roles in hypertension. Despite the negative impact on health described above, fructose gained popularity due to its greater sweetness and low cost [9,10]. Thus, fructose consumption rate has increased due to greater consumption of processed food containing HFCS.

Despite an identical chemical formula to that of glucose, the chemical structure of fructose imparts unique properties that impact its absorption, metabolism and excretion. For example, fructose promotes sodium and chloride absorption by the gut and reabsorption by the kidney. When combined with a high salt diet, fructose leads to a positive net sodium balance and higher blood pressure [11,12,13,14,15,16,17,18]. Fructose intake also excites the sympathetic nervous system which is a key factor for hypertension. These effects are not observed with glucose [18,19,20]. Several transporters and co-transporters for glucose and fructose are highly expressed in the small intestine and kidney, respectively. GLUT5 (also known as Slc2a5) is the primary transporter promoting fructose absorption by the small intestine [13]. The sodium glucose-linked transporter family (SGLT) is another set of transporters with SGLT4 and SGLT5 (also known as Slc5a9 and Slc5a10, respectively) co-transporting fructose with sodium [21]. SGLT4 and SGLT5 are highly expressed in the small intestine and proximal tubule of the kidney, respectively [22]. A very recent study demonstrated SGLT5 mRNA expression in the heart in rats [23]. In contrast to genetic silencing of SGLT4, constitutive knockout of SGLT5 prevents high salt intake from increasing blood pressure, whereas genetic silencing of SGLT4 does not [24,25,26]. At this time, the mechanisms whereby SGLT4 and SGLT5 function differentially to contribute to elevated blood pressure have not yet been fully identified. Nonetheless, SGLT5 may be a potential target for therapeutic approaches addressing salt-sensitive hypertension.

It is well established that a high fructose diet induces salt-sensitive hypertension [27], yet the mechanism for this effect remains incompletely understood. Fructose consumption impedes renal nitric oxide production which augments salt retention, eventually increasing extracellular fluid volume that is not mitigated fully by counterregulatory mechanisms, thus inducing salt-sensitive hypertension [18,28]. Although excess fructose intake alters several cardiovascular parameters [8,29], there are no studies regarding the mechanisms by which SGLTs play a direct role.

We conducted the present review to clarify the mechanism of SGLT4 and, particularly, SGLT5 on fructose reabsorption leading to elevated blood pressure. Hopefully, this will pave the way for a better understanding of SGLT5-associated fructose transport resulting in elevated blood pressure and its potential as a therapeutic target for salt-sensitive hypertension.

## 2. Molecular Structure and Function of SGLTs

The SGLTs are members of the Slc5 transporter family a subset of the APC superfamily of transporters, which includes transporters for sugars, vitamins, glucose, amino acids, fatty acids, and anions [30]. Among the Slc5 family, SGLT1, SGLT2, SGLT4, SGLT5, and SGLT6 transport carbohydrates. In addition, SGLT3 works as a glucose sensor [31]. SGLT6, also known as sodium/myo-inositol transporter 2 (SMIT2), cotransports sodium-myoinositol rather than glucose [32]. The SGLTs are co-transporters that promote carbohydrate transport into the cell secondary along with sodium [33,34]. The energy to transport the monosaccharide is generated from Na^+^ moving down its electrochemical gradient established by the action of Na^+^K^+^-ATPase [30]. SGLT1 as a high-affinity, low-capacity transporter plays a crucial role in glucose absorption in the small intestine and less so in the renal proximal tubule [30]. In contrast, SGLT2 has low affinity but high capacity for reabsorbing glucose from glomerular filtrate back into the bloodstream [35].

The specific structure of each of the human SGLTs has not been fully elucidated. Much of what is known is based on the crystal structure of vSGLT1 [36], the Na^+^-galactose transporter of Vibrio parahaemolyticus that bears significant homology with the mammalian symporters [37]. Cryo-EM studies have identified key features of the molecular structures of SGLT1 and SGLT2 in the outward-open, occluded and the inward-open conformations in the substrate-bound state [38,39]. Crystallographic of cryo-EM studies of SGLT4 or SGLT5 have yet to be accomplished.

**Figure 1:**
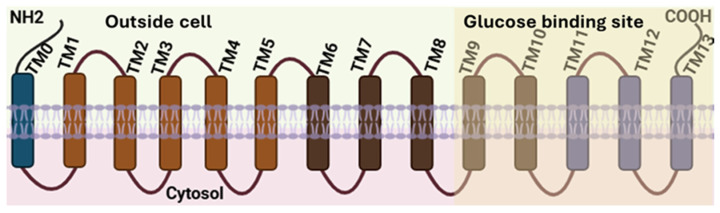
Generalized secondary structure of SGLTs. The SGLT1 664-amino-acid protein contains 14 transmembrane helical domains. Note that in SGLT2, TM1 and TM6 possess breaks in their helical structure (not depicted here).

SGLT1, which couples the transport of either glucose or galactose with Na^+^ ions at a 1:2 stoichiometry was the first to be cloned. As with each of the SGLT co-transporters, SGLT1 has a total of 14 transmembrane helices (TM0-TM13) (Figure 1). An amino acid- polyamine-organocation (APC) fold results in TM1-TM5 and TM6-TM10 in an inverted repeat configuration that results in formation of the substrate binding site that permits sodium and sugar molecules to be co-transported into the cell. Overall, the APC-fold facilitates transport of different organic acid across cell membrane as well as regulates nutrient uptake and cellular signaling [40]. One Na^+^ binding site is near TM1 at the helical break while the second Na^+^ binding site results from the conformation of side-chain oxygens from amino acids in TM1, TM5, and TM8 [41]. The structure of SGLT2 also contains 14 transmembrane helices [39]. TM1-TM5 and TM6-TM10 are structurally similar to SGLT1; however, in SGLT2, TM1 and TM6 are each divided into two short helices [39]. Notably, substitution of threonine-395 by alanine in TM8 appears to confer the 1:1 Na: glucose stoichiometry for SGLT2. SGLT4 and SGLT5 also possess 14 transmembrane helices [42,43]. SGLT4 transports Na^+^ together with either glucose, mannose, fructose, or 1,5-anhydroglucitol (1,5-AG) [44]. Current data indicate that fructose binds to SGLT4 as the β-D-fructopyranose isomer (see below) [44]. SGLT5 also transports fructose and 1,5-AG. Recent data indicate that both murine and human SGLT5s have greater affinity for 1,5-AG than SGLT4 [45]. In contrast, SGLT4 transports mannose more effectively than 1,5-AG [45]. Affinity constants for SGLT and GLUT transporters involved in fructose transport are listed in Table 1.

Mutations in SGLT5 that decrease or abolish its transport capability have been reported in humans [45,46,47,48,49]. Although plasma levels of 1,5-AG are lower, it is unknown if transport of fructose itself or the consequences of high-fructose ingestion are altered in individuals with these mutations. Gliflozins which inhibit glucose transport by SGLT2 are now widely used in the treatment of diabetes [50] and in mitigating progression of cardiovascular [51,52] and renal disease [53]. Glucose competes with 1,5-AG leading to low levels of 1,5-AG in the urine under conditions of hyperglycemia [5,6,11,12,14,16,45]. Importantly, gliflozins also reduced transport of 1,5-AG (remogliflozin > dapagliflozin > empagliflozin) by SGLT5. It is unknown whether fructose excretion also increases when gliflozins are administered. Notably, the function of gliflozins to limit transport of 1,5-AG has been exploited to treat hereditary neutropenias that occur in glycogen storage disease type 1b or congenital neutropenia type 4.

**Table 1:** Affinity constants, Km (mM), for SGLT and GLUT isoforms pertinent to fructose.

	SGLT1	SGLT2	SGLT4	SGLT5	GLUT2	GLUT5
**D-glucose**	0.5–1.8	5	7.7	5	17	N
**D-fructose**	N	>100	~100	0.6	76	6
**D-mannose**	N	>100	0.08–0.15	0.4–1.73	125	N
**D-galactose**	6	>100	N	8	92	N

SGLT1 and SGLT2 from [54]; SGLT4 from [42]; SGLT5 from [26,45]; GLUT2 from [55,56]; GLUT5 from [57]; N, minimal to no transport.

## 3. Structure and Function of Glucose and Fructose

**Figure 2:**
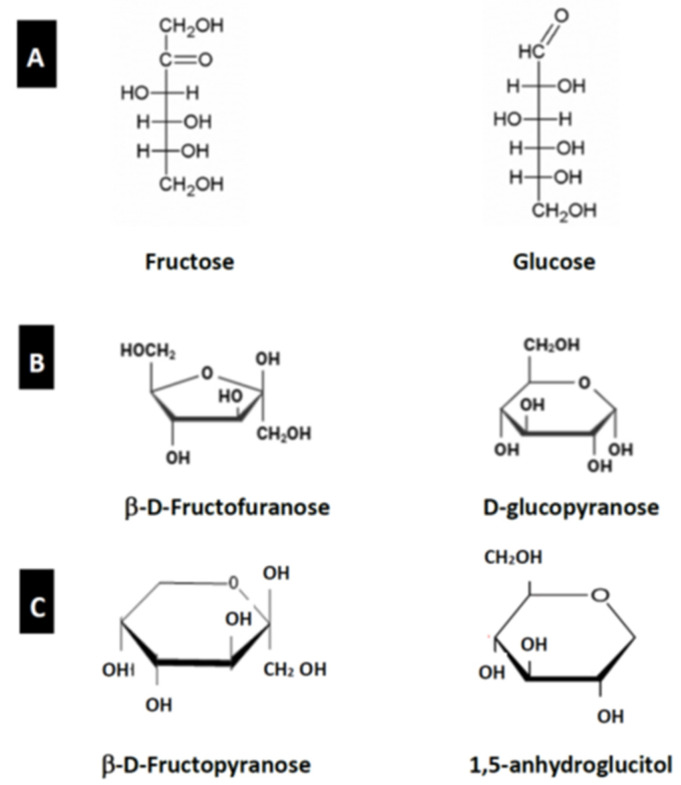
(**A**) Open chain structures for fructose and glucose. (**B**) Ring structures for β-D-fructofuranose and D-glucopyranose which are the favored conformations for binding and transport via GLUT transporters. (**C**) Ring structures for β-D-fructopyranose which is the favored conformation for transport by SGLTs tested thus far and 1,5-anhydroglucicol (1,5-AG) (see text for details).

Glucose and fructose both have the same 6-carbon chemical formula C_6_H_12_O_6_ and are structural isomers. D- and L-isomers exist for both monosaccharides, but it is the D-isomer that is utilized by cells for energy. Glucose has an aldehyde group where fructose contains a functional ketone group (Figure 2A) [58]. Although the chemical composition of glucose and the fructose is the same [59], fructose is a 5-ring structure compared with the 6-carbon ring structure of glucose. Fructose may exert its negative impact on health in part due to the presence of a functional keto group on carbon-2 [60,61,62]. This difference in chemical structure directs fructose into a less regulated metabolic pathway that differs from that of glucose and results in increased fat production and less negative feedback via satiety pathways.

Glucose cyclizes into a six-membered pyranose ring (aldohexose), while fructose forms a five-membered furanose ring (ketohexose) while that of fructose is a 5-member hemiketal, or ketohexose ring [63]. Within the aqueous environment of the circulation, the ring structures of glucose and fructose differ substantially with glucose existing as a hexamer (pyranose) and fructose as a pentamer (furanose (Figure 2B); however, fructose can also exist in its pyranose form as fructopyranose (Figure 2C). The pyranose structures of glucose and fructose differ due to the specific carons that result in the ring formation and can exist in either α or β anomeric forms [64]. The β-fructopyranose is the dominant moiety. While 99% of glucose exists in its pyranose configuration, fructose is 67.5% pyranose and 31.5% furanose with <1% being in the open chain structure. Studies on GLUT transporters indicate that, at least for GLUT1 and GLUT2, the transporter favors the fructofuranose and glucopyranose conformations [65]. Fewer studies have been performed on the SGLTs. Fructose in its pyranose configuration binds to SGLT1 and SGLT4 but is not transported by SGLT1. Notably, the similar structures of β-D-fructopyranose and 1,5-AG may account for the ability of SGLT4 to transport 1,5-AG (Figure 2C) [44]. Although SGLT4 can transport fructose, its major function in the intestine is to absorb mannose [42]. The bulk of ingested fructose is absorbed from the gastrointestinal tract via GLUT5 which can transport either the furanose or pyranose forms but favors the furanose moiety [66]. Thus, the specific ring structure of fructose may influence how effectively it is absorbed or reabsorbed via different transporters in the gut and kidney [44,67] whose major function is to reabsorb fructose in the kidney.

A thorough discussion of fructose metabolism is beyond the scope of the present summary and has been comprehensively explained by several excellent recent reviews [4,68,69]. In brief, once absorbed from the gastrointestinal tract, fructose is metabolized primarily by the liver which extracts up to 70% of an oral fructose load. Within the liver, fructokinase converts fructose to fructose-1-phosphate which is further catalyzed by aldolase B into glyceraldehyde and dihydroxyacetone phosphate [70]. Unlike glycolytic enzymes involved in glucose metabolism, fructokinase functions independent of insulin or ATP availability. The resulting hexose and triose-phosphate products can then enter pathways for gluconeogenesis, glycogenesis, or lipogenesis depending on the physiological state of the organism. However, fructose can also be metabolized by the intestinal epithelium, adipose tissue, skeletal muscle or kidney. In brief, the kidney has long been known to be capable of gluconeogenesis [71,72]. Although lactate, alanine, pyruvate, and glutamine can all act as substrates, early studies by Krebs and Lund [73] demonstrated that the rate of gluconeogenesis in the renal cortex from fructose is more efficient than several other substrates tested. More recent studies have focused on endogenous fructose production by the kidney and its possible role in acute and chronic kidney diseases [69]. Thus, excess fructose ingestion can increase plasma concentrations up to 10-fold or more [74]. Thus, it is plausible that high fructose diets lead to albeit transient high plasma fructose levels that undergo glomerular filtration and then reabsorption by the proximal tubules via SGLT4 and SGLT5 to enter the fructolytic pathways that may contribute to renal pathology.

## 4. Localization of Different SGLTs

**Figure 3:**
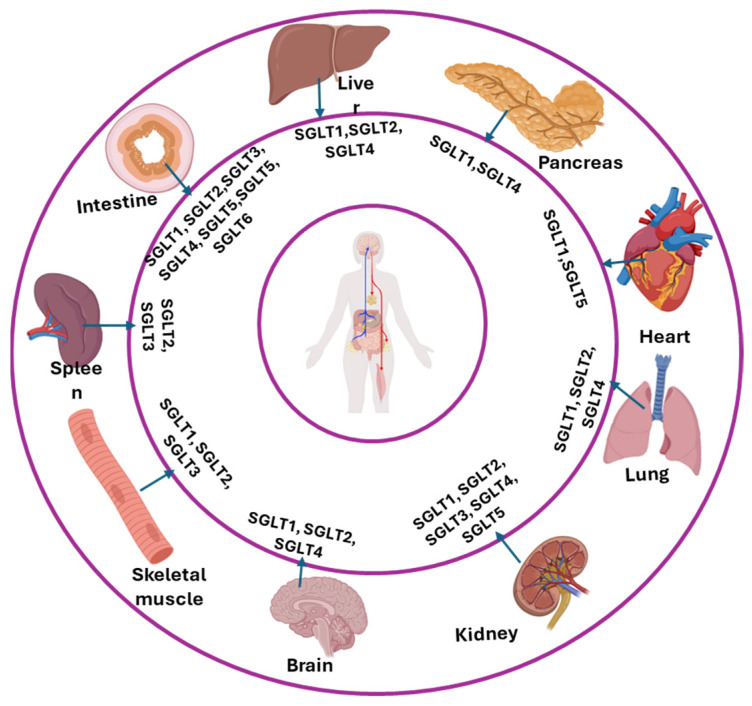
Localization of different SGLTs in different organs of the body.

The SGLTs are members of the larger APC superfamily of transmembrane proteins which transport nutrients, such as glucose, amino acids, and vitamins in different organs of the body including small intestine, kidney, lung, heart, and others. The SGLTs cotransport monosaccharides together with sodium via secondary active transport. Along with the GLUT transporters, SGLTs primarily function to permit absorption of ingested sugars and retrieval of filtered sugars by the renal tubules. In other tissues, the SGLTs contribute to sugar transport into cells for energy utilization and other metabolic processes such as lipid metabolism. To date, a total of six isoforms of SGLTs have been reported in humans (Table 2, Figure 3) [75]. Of the SGLTs, the SGLT1 and SGLT2 transporters have been studied more thoroughly, but information about other members of the transport family is rapidly accumulating.

**Figure 4:**
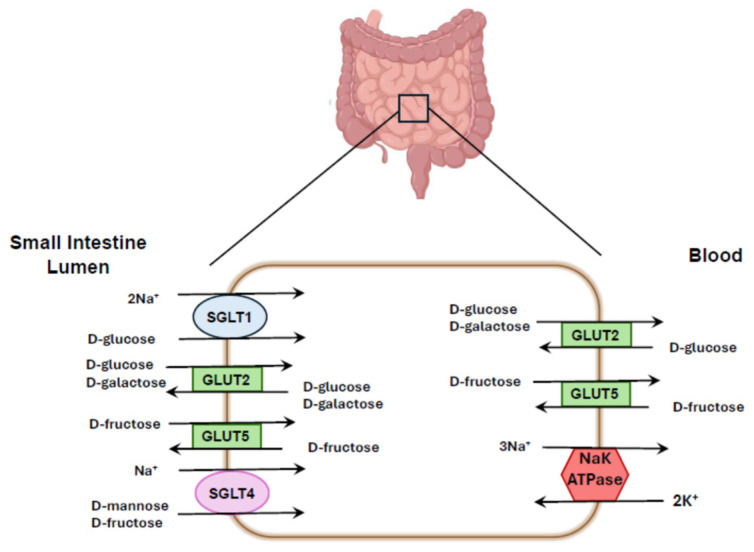
Glucose transport by SGLTs and facilitated by GLUTs in the intestine. Fructose is transported from the gut lumen into the cell primarily via GLUT5 on the apical membrane and then into the interstitium and circulation via GLUT5 on the basolateral membrane. GLUT5 can transport bidirectionally. In the intestine, SGLT4 can transport fructose but favors the transport of mannose along with Na^+^ in 1:1 ratio. Glucose enters the gut epithelium either through GLUT2 or SGLT1. The latter transports glucose in 2:1 ratio. Glucose is absorbed into the circulation after transport into the interstitium by GLUT2 on the basolateral membrane.

SGLT1 (SLC5A1) is localized in the small intestine, the S3 segment of the renal proximal tubule, salivary gland, liver, lung, skeletal muscle, heart, brain, and α-cells of the pancreas in both humans and animals [76,77,78,79]. Robust expression of SGLT1 has been demonstrated in human small intestine and in human proximal tubules [77]. Notably, renal SGLT1 expression in mice depends not only on sex (males > females) but also on the tubular segment involved (S2 vs. S3). This was not observed in rats for SGLT1 nor in other organs [78]. In the small intestine, glucose is primarily absorbed via GLUT2 and SGLT1 on the luminal membrane, whereas fructose is absorbed via GLUT5 and SGLT4 in both humans and rats (Figure 4) [31,80,81]. Quantitative PCR using a primer from exon 6–7 revealed SGLT2 mRNA is localized primarily in kidney cortex. This finding was confirmed by using a primer designed from exon 13 which also showed sparse expression in a number of other organs [23] in healthy humans. SGLT2 (SLC5A2) protein is expressed abundantly in the S1 and S2 segments of renal proximal tubules of both humans and animals [23,31,82,83,84]. Although protein expression in healthy humans has been primarily found in the kidney cortex, SGLT2 protein has also been demonstrated in duodenum, mammary glands, testis, liver, lung, skeletal muscle, spleen and brain of patients with diabetes or liver disease [85,86]. Whether SGLT2 is expressed in these tissues in normal individuals remains to be studied. SGLT3 (SLC5A4) is found in the small intestine, spleen, liver, kidney, skeletal muscle and cholinergic neurons. Current data suggest that SGLT3 is nonfunctional as a transporter [87,88] and acts primarily as a glucose sensor [89].

**Figure 5:**
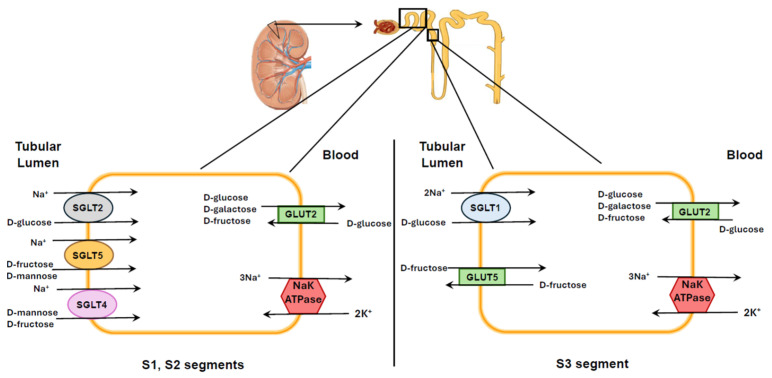
Glucose transport by active SGLTs and facilitated GLUTs in kidney. The early proximal tubule (segments S1 and S2) possesses SGLT2, SGLT4 and SGLT5 on the apical membrane. Glucose enters the cell primarily via SGLT2 in 1:1 ratio with Na^+^. The major transporter for fructose in this segment is SGLT5 although SGLT4 may also have a role. SGLT4 and SGLT5 favor mannose and fructose, respectively. Once in the cell, glucose or fructose can exit the cell via GLUT2 since GLUT5 has not yet been identified in the kidney. In the S3 segment or proximal straight tubule, SGLT1 is the primary carbohydrate transporter which reabsorbs glucose along with 2 Na^+^ ions at the apical membrane; glucose is then transported into the interstitial fluid and thereafter into bloodstream by GLUT2 on the basolateral membrane.

Fewer studies have been conducted on SGLT4, SGLT5, and SGLT6. The SGLT4 (SLC5A9) has been localized in the small intestine, kidneys, liver, lung, brain, trachea, uterus and pancreas [23,31,42,43,79,90]. SGLT5 (SLC5A10) protein was identified only in kidney and testes [26]. However, a very recent study revealed that the SGLT5 mRNA has also been expressed in the heart [23]; however, SGLT5 protein has yet to be demonstrated in cardiac tissue. SGLT5 is responsible for the fructose reabsorption from the kidney in the S1 and S2 segments (Figure 5) [22]. GLUT5 is not present on cells of the S2 segment [91], whereas in the S3 segment, fructose transport at the apical membrane of the proximal tubule is facilitated by GLUT5 [92]. Fructose exits these cells via GLUT2 on the basolateral membranes [91]. Human SGLT6 (SLC5A11) is expressed in the brain and small intestine but has low affinities for either glucose or fructose transport [23,32,93]. The relative affinities for glucose and fructose of the SGLTs are listed in Table 1.

**Table 2:** Summary of localization of different SGLTs in various organs of the body.

SGLTs	Location	Protein/mRNA	Authors	Reference
**SGLT1** **(Slc5a1)**	Intestinal epithelial cell	Protein/mRNA	Vrhovac et al., 2015	[77]
	S3 segment proximal tubule	Protein/mRNA	Vrhovac et al., 2015	[77]
	Salivary gland	Protein	Sabino-Silva et al., 2013	[76]
	Liver	Protein/mRNA	Vrhovac et al., 2015, Liang et al., 2020	[77,79]
	Lung	Protein/mRNA	Vrhovac et al., 2015	[77]
	Skeletal muscle		Madunić et al., 2017	[78]
	Heart	Protein/mRNA	Vrhovac et al., 2015, Liang et al., 2020	[77,79]
	Brain	mRNA	Madunić et al., 2017	[78]
	Pancreatic α-cells	mRNA	Madunić et al., 2017	[78]
**SGLT2** **(Scl5a2)**	S1,S2 segments proximal tubule	Protein/mRNA	Ghezzi et al., 2018	[83]
	Mammary glands	mRNA	Zhao et al., 2005	[82]
	Testis	Protein	Kosinski et al., 2024	[84]
	Liver		Wright et al., 2011	[31]
	Lung	mRNA	Chen et al., 2010	[23]
	Intestine,	mRNA	Chen et al., 2010	[23]
	Skeletal muscle	mRNA	Wright et al., 2011, Zhao et al., 2005	[31,82]
	Spleen	mRNA	Zhao et al., 2005	[82]
	Cerebellum	mRNA	Wright et al., 2011	[31]
**SGLT3** **(Scl5a4)**	Intestine	mRNA	Soták et al., 2021	[88]
	Spleen	mRNA	Diez-Sampedro et al., 2003	[87]
	Kidney	mRNA	Diez-Sampedro et al., 2003	[87]
	Skeletal muscle	Protein/mRNA	Diez-Sampedro et al., 2003	[87]
	Cholinergic neurons	mRNA	Diez-Sampedro et al., 2003	[87]
**SGLT4** **(Scl5a9)**	Small intestine	mRNA	Chen et al., 2010; Tazawa et al., 2005	[23,42]
	Kidneys	mRNA	Tazawa et al., 2005, Liang et al., 2020	[42,79]
	Liver	mRNA	Liang et al., 2020	[79]
	Lung	mRNA	Wright et al., 2011	[31]
	Skeletal muscle	mRNA	Chen et al., 2010, Liang et al., 2020	[23,79]
	Brain	mRNA	Liang et al., 2020	[79]
	Trachea	mRNA	Tazawa et al., 2005	[42]
	Pancreas	mRNA	Gatto et al., 2020	[94]
**SGLT5** **(Scl5a10)**	S1,S2 segment proximal tubule	Protein/mRNA	Chen et al., 2010; Grempler et al., 2012; Fukuzawa et al., 2013; Gonzalez-Vicente et al., 2019	[22,23,26,91]
	Heart	mRNA	Chen et al., 2010	[23]
**SGLT6** **(Scl5a11)**	Small intestine	Protein	Baader-Pagler et al., 2018	[93]
	Brain	Protein	Baader-Pagler et al., 2018	[93]

SGLT. Sodium-dependent glucose cotransporters.

## 5. Fructose, Salt-Sensitive Hypertension, SGLT4 and SGLT5

The SGLTs and GLUTs work together on glucose transport into blood circulation in both small intestine and kidney [95]. Numerous studies have identified the mechanisms whereby SGLT1 and SGLT2 transport glucose and sodium across apical membrane of small intestine and proximal tubules of the nephron to maintain energy balance (reviewed in [31,83,96]). Interest in these transporters heightened with the development of gliflozins, pharmacologic inhibitors of SGLT2, whose utility extends beyond glycemic control to hypertension [97], cardiac and renal protection [90,98,99,100].

Fructose has been implicated in several pathologies such as dyslipidemias, nonalcoholic fatty liver disease, colitis, insulin resistance, malignancies as well as cardiometabolic and renal diseases [70]. High fructose diet is associated with 9% greater risk of cardiovascular disease [101,102] and a 40% greater risk of albuminuria [103], an early index of renal damage. Several investigators have reported an association between fructose consumption and hypertension, particularly when combined with high salt intake in humans [14,21,27,101,104]. Not all studies have observed elevated blood pressure with fructose intake, and it is unclear if concurrent development of frank metabolic syndrome is a requirement [105]. Of interest, a meta-analysis of several cohort studies reported that fructose intake as sugar-sweetened beverages is associated with hypertension but not fructose ingested from other sources such as fruit or dairy products [106]. Studies in rodents have shown a strong association between elevated blood pressure and combined fructose and sodium chloride intake. Positive sodium balance, augmented renin-angiotensin mechanisms, and sympathetic hyperactivity have all been implicated in the mechanism [17,20,29,107].

**Figure 6:**
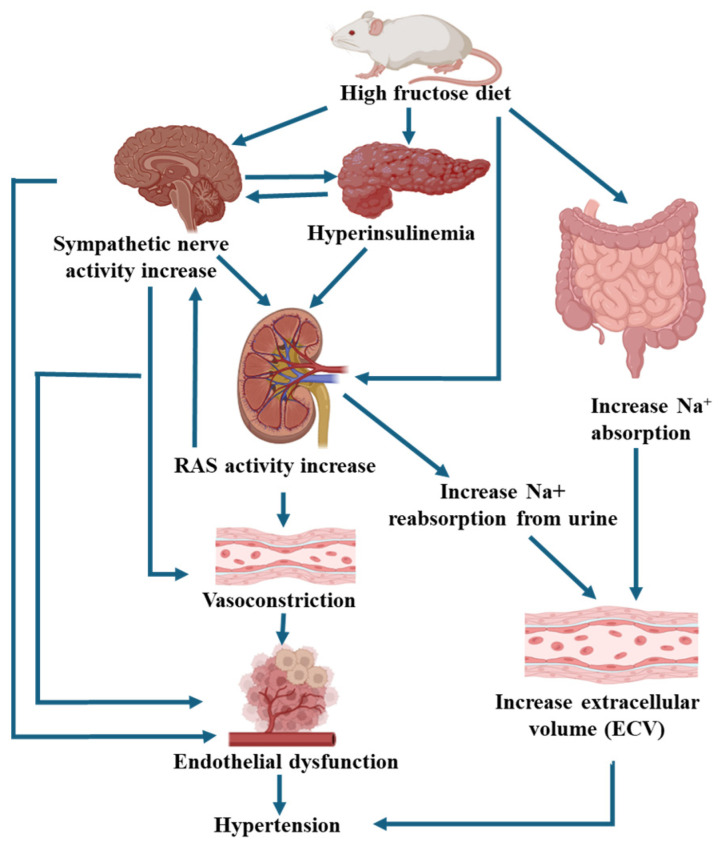
Mechanisms involved in fructose-associated salt-sensitive hypertension.

An increase in fructose intake augments the amount of GLUT5 and SGLT1 in the intestine in tandem with increased expression of the sodium-hydrogen exchanger (NHE3) [12] and the putative anion transporter (PAT1) [11]. Thus, the capability for both fructose and sodium absorption by the gut is enhanced. Consistent with a role for fructose, knockout of GLUT5 prevents the elevation in systolic blood pressure observed with high fructose high-salt diet [12]. In addition, fructose increases the activity of NHE3 [108], and the sodium phosphate cotransporter [109] in the proximal tubule thereby augmenting the ability of the kidney to reabsorb sodium as well. The effect of fructose may be offset, in part, by a decrease in surface expression of the Na^+^K^+^2Cl^−^ cotransporter (NKCC2)in the thick ascending limb [110], an effect that is intriguingly downregulated by aldolase B enzyme in the fructolysis pathway [111]. Nonetheless, a diet enriched in fructose and NaCl results in net positive sodium balance and elevated blood pressure [17,18] (Figure 6).

On the other hand, far less information is available regarding the contribution of SGLT4 and SGLT5 transporters and their possible role in fructose-associated elevations in salt-sensitive blood pressure. Urinary fructose excretion increases significantly in the SGLT5 knockout [22]. Emerging data show that knockout of SGLT5 on a Sprague Dawley background prevented the elevation of systolic blood pressure measured by tail cuff plethysmography in both male and female rats after 7-day feeding on a 20% fructose and 3.6% NaCl. In contrast, SGLT4 knockout rats of either sex displayed salt-sensitive hypertension similar to that of wild-type rats [24]. Blood pressure goes down in SGLT5 knockout rat but not the SGLT4 knockout rat is consistent with SGLT4 being primarily a mannose transporter [24,42,44,45]. When measured via direct arterial cannulation under isoflurane anesthesia, rats on a fructose plus high-salt diet identical to that in the study by Forester et al. [24] display higher blood pressure only in the male wild-type Sprague Dawley rats [112]. Interestingly, the latter investigators subsequently confirmed that blood pressure rises equally in male and female conscious, freely moving rats when measured by telemetry [8]. These findings, along with other studies, strongly caution that the impact of type and depth of anesthesia needs to be considered in assessing hemodynamic parameters vis a vis sexual dimorphism, age, and other physiological factors that may affect reproducibility [113,114,115,116].

Rats on a high fructose diet display overall positive sodium balance [17,18]. Available data indicate that gastrointestinal absorption and renal reabsorption of sodium together conspire to result in excess total body sodium. Luminal fructose within the small intestine enhances salt absorption via NHE3 and PAT1 [11,12]. Both the elevated systemic pressure and the positive net sodium balance leading to increased extracellular volume together fail to inhibit renin unless the diet is extended for a longer period of time and, presumably, greater volume expansion [18,20,29]. Moreover, high-fructose, high-salt fed rats exhibit heightened renal sympathetic nerve activity which can also stimulate circulating as well as intrarenal RAS as well as directly regulate proximal tubule sodium reabsorption [117,118,119], and renal denervation improves the elevated blood pressure in fructose-fed rats [20]. Like the jejunum, the presence of fructose in the lumen of the proximal tubule enhances the activity of NHE3 and sensitizes renal sodium reabsorption in response to angiotensin II (Ang II) [16]. Notably, the expression of proximal tubule SGLT5 is increased on a fructose-enriched diet thereby contributing to greater sodium reabsorption [91]. Fructose plus high-salt diet increases Ang II stimulated superoxide formation by proximal tubules in suspension generated from wild-type rats. Genetic silencing of SGLT4 does not alter this response, but knocking out SGLT5 abrogates prevents O_2_^−^ production [24]. Elevated blood pressure in wild-type Sprague Dawley rats was also abrogated by treatment with Tempol, a free radical scavenger [120,121], or losartan, an angiotensin receptor blocker [8,112,121]. These findings support a key role for SGLT5 in the development of renal oxidative stress thereby leading to salt-sensitive hypertension on a high-fructose, high-salt diet. In addition to fructose increasing proximal tubule sodium reabsorption directly by augmented expression of SGLT5 and NHE, sodium transporters downtream nephron segments are also enhanced. Increased surface expression and activity of NKCC2 in the thick ascending limb of Henle has also been reported with high fructose diet [122]. Fructose-fed mice display enhanced phosphorylation of the sodium chloride co-transporter (NCC) in the distal convoluted tubule and evidence of increased sodium reabsorption [123]. Phosphorylated NCC is also elevated in male human subjects given an acute fructose load over 3 h although urinary sodium excretion does not change in this time period [123]. Moreover, the abundance of both total and cleaved epithelial sodium channel (ENaC) on principal cells of the collecting tubules and ducts of mice is increased, and inhibition of ENaC with amiloride prevents the increase in blood pressure in mice fed a high-fructose, high-salt diet [124]. Taken together, these data suggest that a high-fructose diet, especially when combined with high salt intake, augments sodium reabsorption not only by the proximal tubule but throughout the nephron.

Unlike glucose, fructose does not itself stimulate insulin secretion [125], but long-term ingestion of fructose leads to insulin resistance in both animals and humans (reviewed in [126]). A fructose-enriched diet also suppresses leptin and increases ghrelin [127], thereby impairing satiety signals and ultimately leading to obesity. As a result of insulin resistance, circulating insulin levels increase. Insulin stimulates sodium reabsorption by the kidney via NHE3 in the proximal tubule [128], the NKCC2 cotransporter in the thick ascending limb of Henle [129], the thiazide-sensitive cotransporter in the distal tubule [130] and the epithelial sodium channel, ENaC, in the collecting duct [131]. Thus, hyperinsulinemia as well as direct action of fructose along the entire nephron can further enhance sodium reabsorption and contribute to positive sodium balance, volume expansion and ultimately hypertension.

Although the direct mechanisms are not yet fully identified, the oxidative stress resulting from a high-fructose, high-salt diet leads to decreased vascular compliance and left ventricular diastolic dysfunction, cardiac fibrosis, albuminuria and decline in renal function [8,29,120]. Whether these are direct effects of fructose or secondary to elevated blood pressure will require additional studies. The contribution of SGLT4 and, especially, SGLT5 to salt-sensitive hypertension, cardiovascular and renal dysfunction remains to be deciphered.

## 6. Conclusions

Hypertension is a major risk factor for cardiovascular and renal disease. A substantial body of evidence implicates high-salt and high-fructose diets in the development of hypertension. Despite strong evidence-based recommendations to limit intake of these nutrients, consumption remains high. Understanding the function of SGLT4 and SGLT5 on the fructose handling by the body, and the interplay with sodium may provide insights and new therapeutic modalities to address the hypertension and related disorders. Future efforts should continue to focus on effective efforts to diminish fructose content in foods and limit the intake of beverages, condiments and prepared foods high in fructose. In addition, there is a crucial need for continued rigorous investigation of the mechanisms whereby SGLT4 and SGLT5 transport carbohydrates, the metabolism of fructose by different cell types, and the direct and indirect mechanisms whereby these transporters and fructose contribute to salt-sensitive hypertension. The potential development of selective inhibitors, such as those currently available for SGLT2, will also need to address the apparent paradoxical development of hepatic steatosis that occurs in SGLT5 knockout rats.

## 7. Limitations

Thus far, information on the roles of SGLT4 and SGLT5 is accumulating but remains limited. Since there are no specific inhibitors of these transporters, studies have needed to rely on genetic silencing of the transporters. A cautionary note also needs to be raised as contrary to what would be expected, hepatic steatosis was aggravated by knockout of SGLT5 in mice [22]. The complex nature of fructose metabolism will need carefully designed and executed integrated genetic, metabolomic, proteomic and pathophysiologic approaches.

## Abbreviations

The following abbreviations are used in this manuscript:

1,5-AG	1,5-anhydroglucitol
APC	Amino acid-polyamine-organocation
ENaC	Epithelial sodium channel
GLUT	Glucose transporter
NHE3	Sodium-hydrogen exchanger 3
PAT1	Putative anion transporter 1
RAS	Renin–angiotensin system
SGLT	Sodium-dependent glucose linked cotransporter
TM	Transmembrane
SMIT2	Sodium/myo-inositol transporter 2
